# Supplementary material for: Salmonella enterica serovar Typhi uses two type 3 secretion systems to replicate in human macrophages and colonize humanized mice
Source: mBio. 2023 Jun 21;14(4):e01137-23. doi: 10.1128/mbio.01137-23 (PMC10470537; doi:10.1128/mbio.01137-23)
Supplement: Fig S1 — Supplemental data for Figure 1. [file mbio.01137-23-s0005.pdf]

Supplemental Figure S1

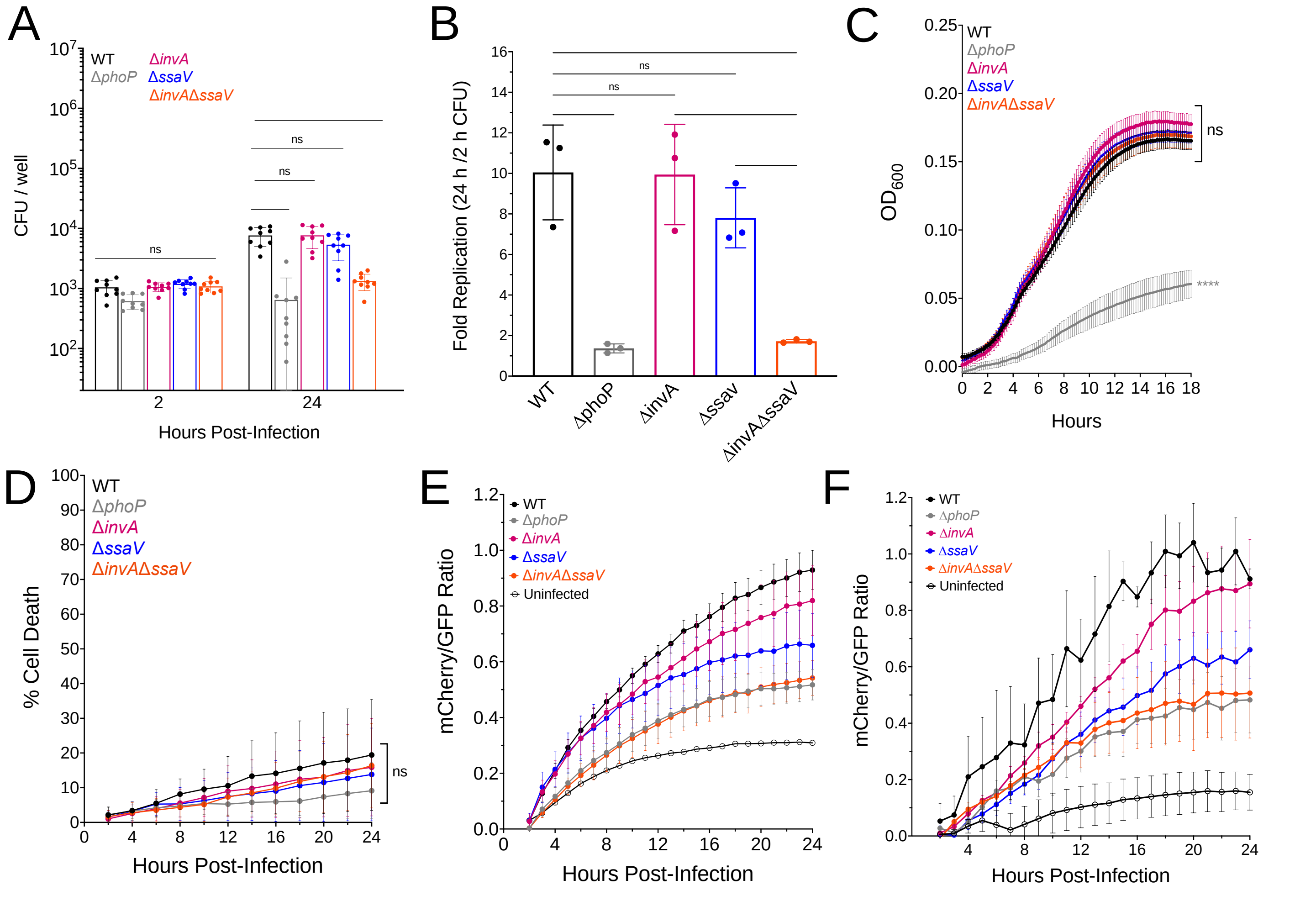

Figure S1. A. Colony forming units (CFU) per well of infected THP-1 macrophages. Statistical significance by ANOVA. Bars: mean. Dots: viable bacteria obtained from each well, 3 wells per 1 biological replicate.

B. Replication of *S.* Typhi in THP-1 macrophages by CFU/well at 2 and 24 h.p.i. Statistical significance by ANOVA. Dots: biological replicates, each an average of 3 technical replicates. Bars: mean. Error: SD.

C. Optical Density (OD) of *S.* Typhi strains in SPI-2-inducing minimal medium (PCN, 0.4 mM KPO_4, pH 5.8). Statistical significance by ANOVA. Line: Mean OD measurement of 3 biological replicates every 15 minutes. Error: SEM.

D. Percent of mCherry+ (Infected) THP-1 macrophages also positive for Sytox green (Dead) based on automated images taken every hour for 32 hours. At 34 h.p.i. 1% Triton-X added to wells to confirm dye presence and to determined 100% lysis. Statistical significance by ANOVA. Line: Mean of 3 biological replicates. Error: SD.

E. Ratio of mCherry/GFP throughout infection by time-lapse microscopy, prior to subtraction of background signal obtained in uninfected wells. Dots: Mean of 6 biological replicates. Error: SEM.

F. Ratio of GFP/mCherry throughout infection by time-lapse microscopy. Data displayed is prior to subtraction of background signal obtained in uninfected wells. Dots: Mean of 4 biological replicates. Error: SEM.

For all; ns = p-value > 0.05, *≤0.05, ** ≤0.01, *** ≤0.001, **** ≤0.0001
