# Supplementary material for: Salmonella enterica serovar Typhi uses two type 3 secretion systems to replicate in human macrophages and colonize humanized mice
Source: mBio. 2023 Jun 21;14(4):e01137-23. doi: 10.1128/mbio.01137-23 (PMC10470537; doi:10.1128/mbio.01137-23)
Supplement: Fig S2 — Supplemental data for Figure 2. [file mbio.01137-23-s0006.pdf]

Supplemental Figure S2

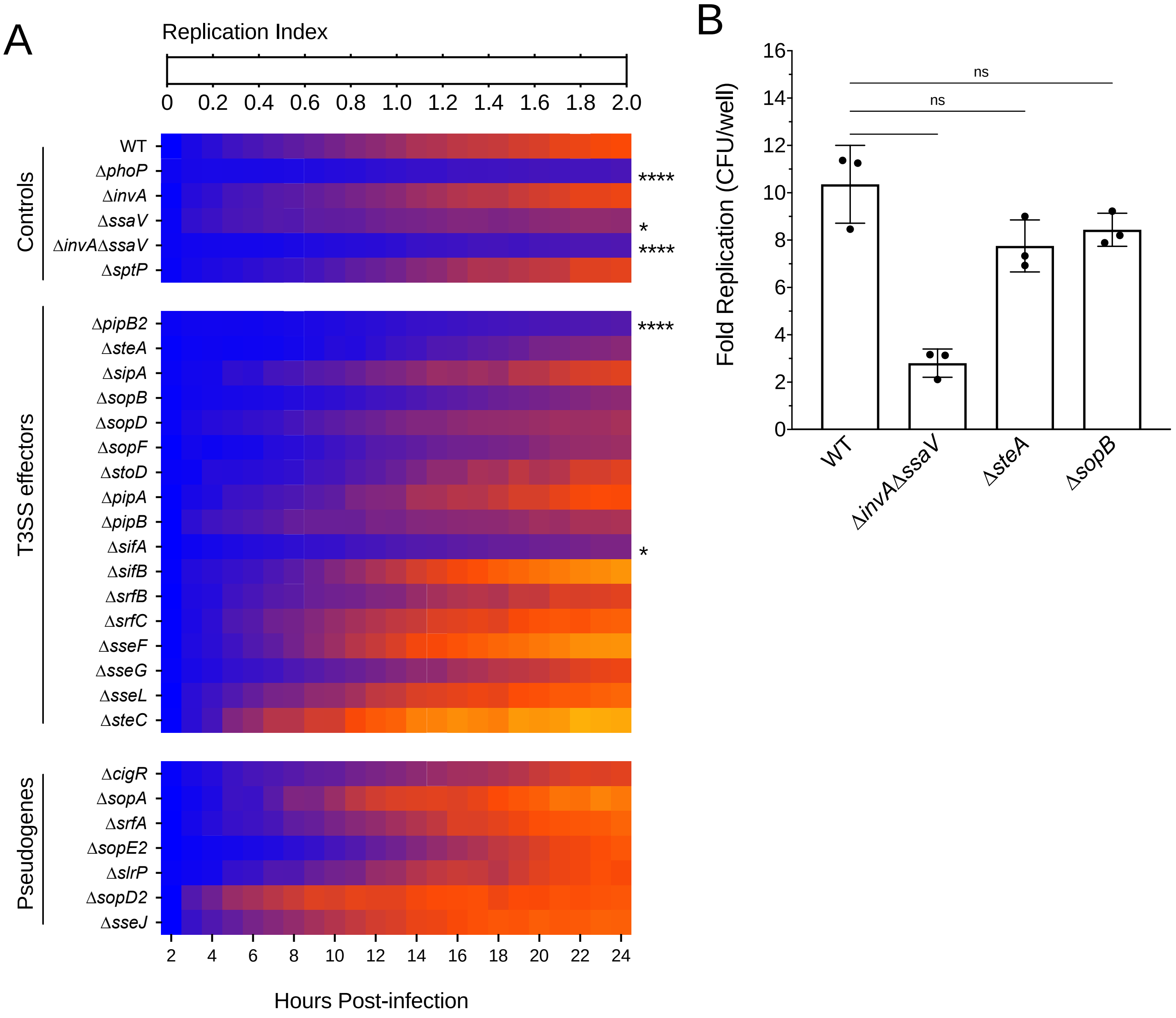

Figure S2. T3SS-dependent effectors contribute to *S.* Typhi replication in THP-1s.
A. Heat map of replication index over 24 hours for each *S.* Typhi strain in THP-1 macrophages by time-lapse florescence microscopy. Each square: mean of 3-5 biological replicates. Statistical significance at 24 h.p.i. compared to hypothetical mean of 1.0 by Wilcoxon test.
B. Replication of *S.* Typhi in THP-1 macrophages by CFU/well between 2 and 24 h.p.i. Statistical significance by ANOVA. Dots: biological replicates, each an average of 3 technical replicates. Bars: mean. Error: SD.
