# Supplementary material for: Salmonella enterica serovar Typhi uses two type 3 secretion systems to replicate in human macrophages and colonize humanized mice
Source: mBio. 2023 Jun 21;14(4):e01137-23. doi: 10.1128/mbio.01137-23 (PMC10470537; doi:10.1128/mbio.01137-23)
Supplement: Fig S3 — Supplemental data for Figure 3. [file mbio.01137-23-s0007.pdf]

Supplemental Figure S3

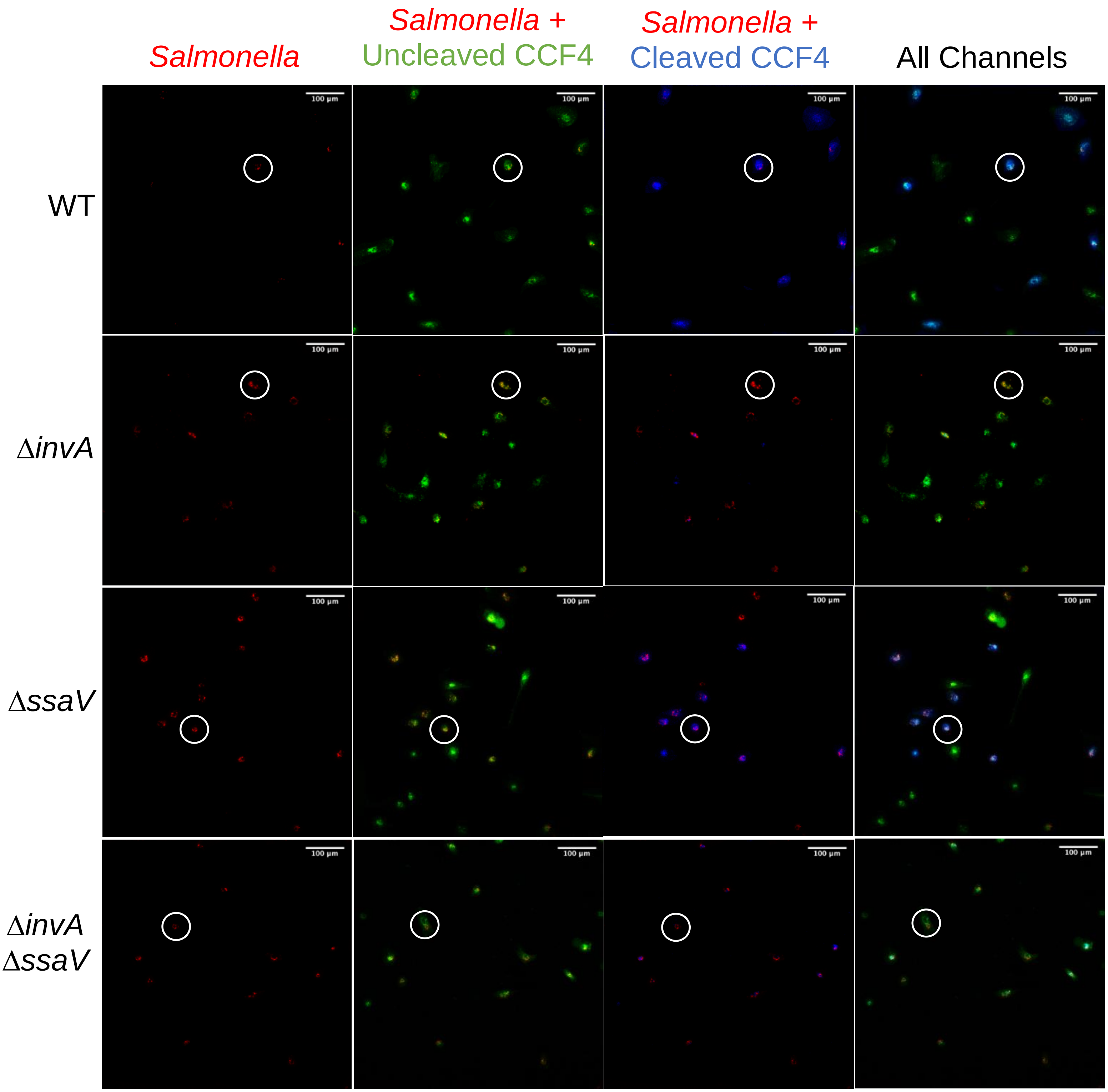


**Figure S3. Represetnative images of SifA-BlaM translocation in hMDMs 8 hours post-infection**
hMDMs infected with Ty2 strains pSifA-BlaM. Infected wells dyed with CCF4, fixed and imaged at 20X, 8 h.p.i. Columns = channels, in order of Red: *Salmonella*, Green: Uncleaved CCF4 dye, indicating hMDM cytosol, Blue: Cleaved CCF4 dye, indicating BlaM in hMDM cytosol, Composite: all three channels overlayed. Rows = *S.* Typhi WT or knock-out strain expressing SifA-BlaM. Scale Bar: upper right. White circles indicate a representative example of one infected hMDM in each channel and the composite.
