# Supplementary material for: Salmonella enterica serovar Typhi uses two type 3 secretion systems to replicate in human macrophages and colonize humanized mice
Source: mBio. 2023 Jun 21;14(4):e01137-23. doi: 10.1128/mbio.01137-23 (PMC10470537; doi:10.1128/mbio.01137-23)
Supplement: Fig S4 — Supplemental data for Figure 4. [file mbio.01137-23-s0008.pdf]

Supplemental Figure 4

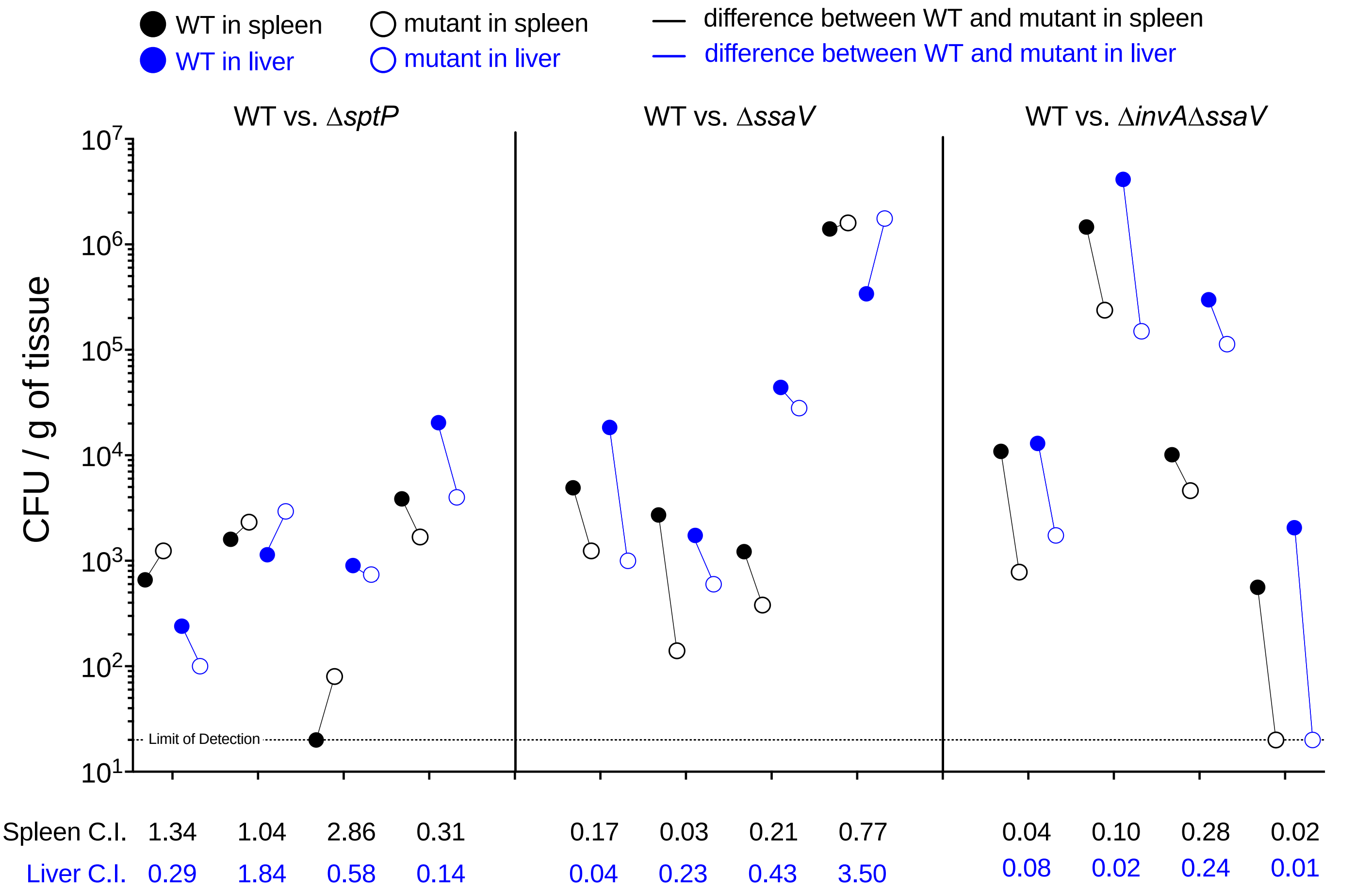

Figure S4. CFU per gram of tissue in spleen and liver of each infected huamized mouse at 5 days p.i. *S.* Typhi burden in whole spleens (black symbols) and livers (blue symbols) sampled for each mouse at 5 days post-infection. Individual mice across X-axis with which two strains were used for IP infection. Y axis indicates CFU per g of homogenized tissue, WT (solid circles) and mutant strain (empty circles) abundance distinguished by kanamycin resistance. A line connects WT and mutant counts from the same organ.
